# Immunopathogenesis of COVID-19: Summary and Possible Interventions

**DOI:** 10.3389/fimmu.2020.564925

**Published:** 2020-09-17

**Authors:** Francois Henri Jacques, Erik Apedaile

**Affiliations:** Clinique Neuro-Outaouais, Gatineau, QC, Canada

**Keywords:** COVID-19, immunology, intervention, hyperinflammation, immunosuppression

## Abstract

Since the onset of the COVID-19 pandemic in the fall of 2019 over 4 million people have been infected and over 280,000 have died (1). Information about the SARS-CoV2 virus is evolving rapidly. At this time there are no interventions proven to be effective for cases infected with SARS-CoV2. Current knowledge about the clinical and laboratory manifestations of COVID-19 infection is reviewed and combined with knowledge about the immunopathogenic mechanisms of Severe Acute Respiratory Syndrome Coronavirus (SARS-CoV1) and Middle East Respiratory Syndrome (MERS) to formulate theories and suggest possible therapeutic interventions. SARS-CoV2 immunopathogenic mechanisms vary from immunosuppression that initially enables viral escape to a hyperinflammatory immune response. Ultimately therapeutic intervention will be phase dependent.

## Introduction

Since the onset of the COVID-19 pandemic in the fall of 2019 over 4 million people have been infected and over 280,000 have died (1). Information about the SARS-CoV2 virus is evolving rapidly. At this time there are no interventions proven to be effective for patients infected with SARS-CoV2. In the majority of SARS-CoV2 infections symptoms remain mild to moderate. According to the Diamond Princess series, 18% remained asymptomatic (2). However, approximately 15% of cases were severe to fatal. Understanding the immunological basis of how the severe cases differed from the asymptomatic cases may form the basis for effective treatment. During an acute infection, the immune system must balance between mechanisms that reduce viral replication and those that prevent immune mediated focal tissue damage. This is akin to driving with one foot on the accelerator while the other is on the brake. In severe SARS-CoV2 infections, the virus appears to first immunosuppress the host, taking advantage of the body’s natural braking system resulting in imunosuppression. This leads to unchecked viremia followed by a hyperinflammatory response manifested clinically as an Acute Respiratory Distress Syndrome (ARDS) and multi organ failure. Depending upon the stage of infection (immunosuppression or hyperinflammation) different interventions may be appropriate, including PD1 inhibitors (stage 1) and plasmapheresis/IL-IB inhibitors/C5 inhibitors (stage 2).

## Immune Response to COVID-19 Infection

### COVID-19 Case Series

Thevarajan (3) reports the immunological profile of a 47-year-old woman, moderately symptomatic with SARS-CoV2 infection requiring hospitalization but no oxygen supplementation. The patient stopped viral shedding on day 7 after symptom onset. Her chest X-ray cleared by day 10 and she became asymptomatic by day 13. Immunologically, at day 7 her lymphocyte and neutrophil counts remained normal. She had an increased C-reactive protein, increased activated CD8+ T cells, reduced levels of CD16+CD14+monocytes, normal levels of NK cells, low levels of chemokine CCL2 and minimal levels of cytokines IL-6, IL-10 MIP-1B, and INF gamma.

Severe cases of SARS-CoV2 infection follow a very different clinical and immunological course. In a retrospective analysis of 499 SARS-CoV2 infected patients, Diao et al. demonstrated the presence of virus induced T cell lymphopenia and T cell exhaustion (4). T cell exhaustion arises during chronic infections and cancers. It is defined by poor effector function, sustained expression of inhibitory PD1, LAG3, CTLA-4, and TIM3 receptors, and a transcriptional state distinct from that of functional effector or memory T cells (4–6). SARS-CoV2 infected patients with poor clinical evolution demonstrate a marked reduction in CD4+ and CD8+ T cells along with a significant degree of T cell PD1 and TIM3 overexpression, indicative of T cell exhaustion. Both lymphopenia and T cell exhaustion were directly correlated with the increase in the levels of IL-6, IL-10, and TNF alpha and with disease severity. The authors surmised that the T cell lymphopenia was mediated by sustained elevation in IL-6, TNF-alpha cytokines and T cell exhaustion from the increased levels of IL-10 (4). In a review of 191 hospitalized cases from Wuhan, China it was noted that the median time from symptom onset to hospitalization, intubation, death and hospital discharge was 11, 14.5, 18.5, and 22 days, respectively (7). Non-survivors compared to survivors had significantly more ARDS (93%), shock (70%), and coagulopathy (50%). Non-survivors also had elevated D-dimers (81%), LDH (98%), ferritin (96%), C-reactive protein (84%), thrombocytopenia (20%), and lymphopenia (40%) (7). In a case series from Seattle of 24 SARS-CoV2 critically ill patients admitted to ICU with ARDS, 75% had hypotension and 77% had lymphopenia. None of the cases had coinfection by other viruses or bacteria. The ratio of increased neutrophils to reduced lymphocytes and the increased level of D-dimers were all predictive of poor outcome (8, 9). Huang et al. demonstrated that the levels of IL-2, IL-7, IL-10, TNF-α, G-CSF, IP-10, MCP-1, and MIP-1A were significantly higher in severe SARS-CoV2 patients (10). In these series of hospitalized patients, older age, obesity, diabetes hypertension, and cardiovascular disease appear to identify patients at a greater risk of poor clinical evolution (7–9).

### Stage 1 – Immunosuppression

The first stage of this apparent overlapping two stage infection is immunosuppression. This is characterized by lymphopenia combined with T cell exhaustion and inadequate adaptive immune response. The objective is to evade the immune system allowing for unchecked viremia. Viruses in general have acquired many immune evasion strategies. SARS-CoV2 is a member of the Beta coronavirus family which are enveloped viruses with a positive sense single-stranded RNA genome. SARS-CoV2 has significant genomic similarity with Severe Acute Respiratory Syndrome Coronavirus (SARS-CoV1) (80%) and Middle East Respiratory Syndrome (MERS) (50%) (8, 11). SARS-CoV1 and MERS have evolved defenses to evade the immune system such as using double membrane vesicles which lack pattern recognition receptors, impairing early production of interferon type 1 and its downstream signaling, increasing early production of inhibitory cytokines such as IL-10 and overexpressing PD-L1 receptors on hematopoietic and non-hematopoietic cells and PD1 on T cells. These viruses can also downregulate antigen presentation by MHC class I and II which diminishes T cell activation and shifts the Th1 response to a Th2 cytokine profile and promotes a T cell exhaustion phenotype (11–13).

Viruses have also evolved strategies to evade the complement system. These include producing proteins that block the complement from binding to the Fc region of antibodies, increased clearance of antibody-antigen complexes from its membrane and production of complement inhibitory proteins that bind C3b and C4b and serve as cofactors for factor I thus preventing complement activation at the virus membrane (14).

### Stage 2 – Hyperinflammation

The second stage in severe SARS-CoV2 infection is characterized by a cytokine storm with neutrophil, monocyte/macrophage infiltration and activation. This is manifested clinically by ARDS, multi organ failure and coagulopathy. Complement activation though not yet confirmed likely plays a role. All three parts of the complement system are activated in mice models of SARS-CoV1. The infected wild type mice model shows increased level of cytokines (IL-1, IL-6 TNF alpha, G-CSF), chemokines, increased numbers of neutrophils and monocytes as well as evidence of complement deposition in mice lungs. There is also evidence of prominent serum activation of complement mediated systemic inflammation. The SARS-CoV1 infected C3 knock out mice model showed significant dampening of the inflammatory parameters and less weight loss by the mice despite a lack of difference in viral titers (15). C5a levels were predictive of ARDS development in patients with SARS-CoV1 (15).

C5a blockade was shown to be protective in the MERS-CoV and H5N1 influenza virus induced acute lung injury mice models (15–17).

## Discussion

### Links Between Unchecked Viremia and Hyperinflammation

**FIGURE 1:**
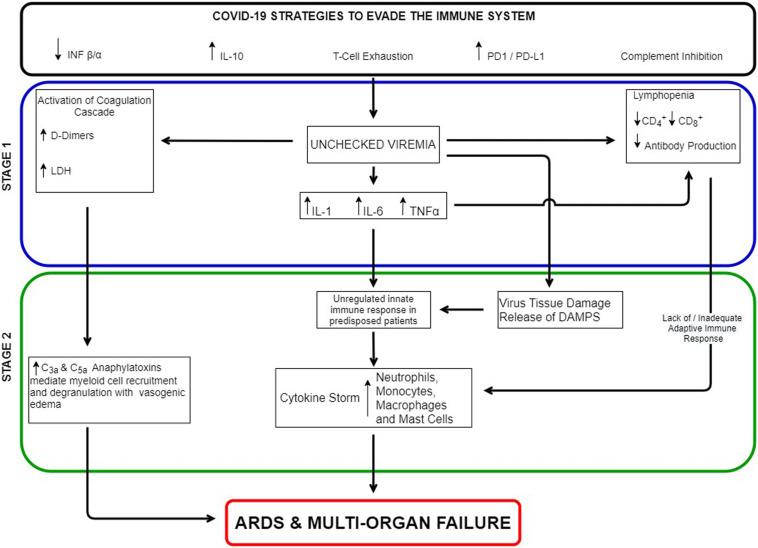
COVID 19 Links between immunosuppression and hyperinflammation.

The links between immunosuppression and hyperinflammation are illustrated in Figure 1. Possible links between unchecked viremia and inflammation based ARDS include virus induced extensive alveolar damage and release of Damage Associated Molecular Patterns (DAMPS) (directly activating the innate immune system), over activation of the complement system’s anaphylatoxins, a lack of an adequate adaptive immune response, an exaggerated innate immune response in predisposed individuals, or a combination of all four. In direct sepsis induced ARDS, pulmonary pathogens activate a robust innate immune response in epithelial cells and alveolar macrophages followed by neutrophil infiltration and monocyte recruitment (18). The release of cytokines such as TNF alpha, IL-1B, and IL-6 leads to loss of alveolar-capillary barrier integrity resulting in secondary alveolar edema (18, 19). Studies have also shown prominent activation of the complement system in ARDS. C3a and C4a levels have been linked to its development. The extent and degree of complement activation has also been correlated with clinical outcome (16).

Among its many functions, the complement system serves as the bridge between the innate and adaptive immune systems. Complement enhances antigen uptake by follicular dendritic cells and via the C5a receptor on Antigen Presenting Cells (APC), influences their cytokine production to a Th1 phenotype. Via the C2a receptor on B cells, complement enhances the T cell dependant antigen specific B cell response. The complement system is also involved in T cell maturation, in enhancing CD4+ and CD8+ T cell responses (14, 20), and in maintaining innate self-tolerance (20). Virus inhibition of the complement system could therefore represent a mechanism by which the virus could hinder the development of the adaptive immune response. However, the anaphylatoxins C3a and C5a fragments appear to be unaffected by virus immune evasion strategies.

The complement system can be stimulated by viral activation of the coagulation cascade. More than 80% of severe SARS-CoV2 infection had elevated D-dimers and 50% have a coagulopathy. Coagulation pathway proteins such thrombin and plasmin can cleave C3 and C5 with the release of the anaphylatoxins C3a and C5a. Platelet consumption in the formation of thrombus also releases ATP and Ca along with serine/threonine kinases. These phosphorylate C3b which protects it from degradation thus further enhancing the complement pathways (14, 20, 21).

Ahmadpoor and Rostaing have proposed that when the body is unable to mount an effective adaptive immune response, persistent innate induced inflammation can become dysregulated leading to a cytokine storm, neutrophilia, and hyperinflammatory monocytes all resulting in ARDS and multi organ failure (22). In elderly patients with less naïve T-cells there is a diminished ability to respond to new pathogens compared to younger immune systems. This could explain the increased mortality seen in older SARS-CoV2 patients.

The identified risk factors of obesity, diabetes and cardiovascular disease may be indicative of an underlying metabolic syndrome. It is hypothesized that increasing serum fatty acids associated with metabolic syndrome stimulate TLR4 leading to increased serum levels of TNF-alpha, IL-1B, and IL-6. These cytokines inhibit the downstream signaling of insulin receptors causing insulin resistance, diabetes, and atherosclerosis. Adipocytes themselves can secrete these inflammatory cytokines. Visceral adipose tissue can also secrete leptin which leads to increased levels of INF-gamma (14). Anti-inflammatory therapy targeting the interleukin-1β innate immunity pathway with canakinumab was shown to significantly lower the rate of recurrent cardiovascular events independent of lipid-level lowering (23). Thus, individuals with metabolic syndrome may be more susceptible to a SARS-CoV2 induced cytokine storm and subsequent ARDS.

### Stage 1 Interventions

Possible therapeutic interventions will depend on where in the immunopathogenic process the intervention is applied. Early interventions that look at non-specifically augmenting the Th1 immune response, such as with the BCG vaccine, or specifically circumventing the viral immunosuppressive strategies with an anti PD1(nivolumab, pembrolizumab) or PD-L1 monoclonal antibody (durvalumab, avelumab) may be considered. Animal models have demonstrated that early blocking of the PD1/PD-L1 axis increases the effector function of CD8+ T cells by enhancing granzyme B expression and mTOR signaling (5). Anti PD1 therapy in animal models of HIV and TB showed a reduced hyper activation of interferon type 1 responses resulting in a lower incidence of cytokine storms and immune dysregulation (24). Early intervention with an anti PD-L1 therapy in acute severe SARS-CoV2 infection could tip the balance in favor of increased early viral clearance. Anti PD1/PD-L1 immunotherapy can be associated with immune related adverse effects. These are however treatable and often transient. They can occur even after a single dose but the latter is uncommon (25). Only one or two doses would be required in treating SARS-CoV2 infection. Limiting its use to higher risk patients could further mitigate the risk/benefit ratio.

The BCG vaccine is used to vaccinate neonates in most of the world for tuberculosis. The duration of immunity lasts from 15 to 60 years post immunization. It confers non-specific Th1 immunity and has shown cross over immunity for both tuberculoid and lepromatous forms of leprosy and as an effective immunotherapeutic agent against a variety of cancers such as bladder cancer and melanoma. BCG activates TLR2 and TLR4 on macrophages and dendritic cells which produces IL-1B, IL-12, and TNF-alpha. Antigen presenting cells present via MHC class II to CD4+ T helper cells and produces activated central memory T cell Th1 phenotype with increased IFN gamma and IL-2 (26–28). It is plausible that the use of BCG in the context of recent acute SARS-CoV2 infection could help enhance the initial anti-viral Th1 response and mitigate the risk of further complications. One could also speculate as to the role of BCG vaccination policies in exploring the differences in the impact of SARS-CoV2 infection between countries with BCG vaccination programs and those without. Vaccination with BCG could be used prophylactically in front line workers and at risk populations or soon after an unprotected exposure to SARS-CoV2.

### Stage 2 Interventions

Once a hyperinflammatory state is established different strategies can be attempted to mitigate the immune mediated tissue damage. Plasmapheresis will reduce levels of cytokines, complement and coagulation factors. It is the recommended treatment in many autoimmune diseases such as thrombotic thrombocytopenic purpura (29). Canakinumab is a human monoclonal anti-human IL-1B antibody. IL-1B is the circulating form of IL-1 that depends on the inflammasome to convert it from its precursor to its active form. It is located upstream to other major innate immune system cytokines such as TNF-alpha, IL-6, and IL-12. The CANTOS study confirmed the role of inflammation in atherosclerosis and the impact of canakinumab on coronary disease (23). Such therapy could potentially be useful in patients with comorbidities/metabolic syndrome to prevent the cytokine storm. Complement, especially in patients with concomitant coagulopathy may play an important role in ARDS pathophysiology. Eculizumab is a recombinant humanized monoclonal IgG2/4κ antibody that specifically binds to the complement protein C5 with high affinity, thereby inhibiting its cleavage to C5a and C5b and preventing the generation of the terminal complement complex C5b-9 and free C5a (30). One or more treatments could be useful in dampening immune mediated tissue damage.

## Conclusion

Human infection with SARS-CoV2 is a very recent evolutionary phenomenon. In many cases the virus is very successful in infecting, replicating and spreading to others with little or no symptoms to its host. In a minority of cases, the virus overzealously applies the brakes to the immune system. This triggers a reckless and unfocused innate immune acceleration with detrimental results to itself and its host. If and until a vaccine is developed, interventions will need to be stage dependant and guided by patient clinical characteristics and immune parameters such as levels of lymphopenia, neutrophilia, D-dimers and cytokines. It also remains to be elucidated how such interventions will affect the subsequent development of SARS-Cov2 immunity. The risk/benefit equation for any intervention should therefore be measured not only at the individual but also at the societal level. Clinical trials testing such hypotheses are warranted.

## Data Availability Statement

All datasets presented in this study are included in the article/supplementary material.

## Author Contributions

FJ was responsible for the content. EA was responsible for editing, formatting, and graphics. Both authors contributed to the article and approved the submitted version.

## Conflict of Interest

FJ receives or has received funding from Sanofie, Roche, Merck Serono, Biogen, and Novartis for clinical trials in multiple sclerosis, Parkinson’s disease, Alzheimer’s disease, and Glioblastoma Multiforme. Funding was not sought for this article nor was it reviewed by any organization related in any way to the interventions discussed. The remaining author declares that the research was conducted in the absence of any commercial or financial relationships that could be construed as a potential conflict of interest.
